# The Additional Effect of Autologous Platelet Concentrates to Coronally Advanced Flap in the Treatment of Gingival Recessions: A Systematic Review and Meta-Analysis

**DOI:** 10.1155/2019/2587245

**Published:** 2019-07-25

**Authors:** Rong Li, Yanqing Liu, Tong Xu, Haijiao Zhao, Jingya Hou, Yun Wu, Dongmei Zhang

**Affiliations:** ^1^Department of Periodontology, School of Stomatology, China Medical University, Shenyang, Liaoning 110002, China; ^2^Department of Oral Biology, School of Stomatology, China Medical University, Shenyang, Liaoning 110002, China

## Abstract

**Background:**

To improve the efficacy of regenerative treatment for gingival recessions, the autologous platelet concentrates (APCs) combined with coronally advanced flap (CAF) have been investigated. However, few studies systematically assess the complementary effect of APCs in periodontal regeneration. The present study aims to evaluate the additional effect of different types of APCs to CAF in the treatment of gingival recessions.

**Methods:**

Electronic databases (EMBASE, MEDLINE, and Cochrane Central Register of Controlled Trails) and relevant journals were searched until May 15, 2019. Only randomized controlled trials (RCTs) in English were included. Outcome variables include root coverage (RC), recession depth (RD), clinical attachment level (CAL), keratinized tissue width (KTW), and gingival thickness (GT). Data were analyzed with Revman5.3. The estimate of effect sizes was expressed as the mean differences and the 95% confidence interval.

**Results:**

8 RCTs involving 170 patients (328 sites) were included. Our meta-analysis indicated RC, RD, CAL, KTW, and GT were better improved in the CAF plus APCs groups than the CAF alone. The subgroup analyses revealed that platelet-rich fibrin (PRF) brought significant improvement in RC, RD, CAL, and GT. Concentrated growth factors (CGF) lead clinic beneficial in CAL, KTW, and GT. No significant effect of platelet-rich plasma (PRP) could be found in any clinical parameters when combined with CAF.

**Conclusions:**

PRF could exert additional effect to CAF; the preferred treatment for gingival recessions was considered. Based on the limited studies, it seemed that PRP failed to show any additional effect and it was not suggested for gingival recessions. Given the limited research and high risk of bias, it is still needed to confirm the additional effect of CGF by more high-quality studies.

## 1. Introduction

Gingival recession is a common, undesirable problem attribute to the apical migration of the margin tissue beyond the cemento-enamel junction (CEJ). There is a higher probability of root caries, loss of attachment, and hypersensitivity in teeth with gingival recession [1]. Various mucogingival procedures have been evolved to obtain root coverage such as free gingival grafts, laterally positioned flaps, or guided tissue regeneration, as well as connective tissue grafting (CTG) and coronally advanced flaps (CAF) [2–4]. CAF is a surgical procedure to shift the gingival tissue coronally on the exposed root surface. It has proven to be an effective and predictable technique because of the optimum root coverage results, the good color blending of the treated area, and the recuperation of the original morphology of the soft tissue margin [5]. What is more, it is convenient and the invasiveness is reduced since graft harvesting is not required in CAF. However, it was reported that root coverage treated with CAF alone is unstable in a long time. The root coverage was 89.0% at 1 month postoperatively and decreased to 58.8% at 6 months [6]. Therefore, CAF is frequently combined with various regenerative materials or biological factors.

In the past several years, autologous platelet concentrates (APCs) had emerged as a potential regenerative material; it can be used alone or with other techniques [7]. APC has been proven to play a pivotal role in soft tissue healing. Their effectiveness lies on the continuous release of multiple cytokines, such as transforming growth factor-*β*1 (TGF-*β*1), vascular endothelial growth factor (VEGF), insulin growth factor (IGF), platelet-derived growth factor-AB (PDGF-AB), and interleukin-1*β* (IL-1*β*) [8]. Until now, three generations of APCs have been developed, including platelet-rich plasma (PRP), platelet-rich fibrin (PRF), and concentrated growth factors (CGF). PRP is the first generation of APCs; it contains a high concentration of platelets obtained by special centrifugation from fresh whole blood. It is required to induce fibrin polymerization by chemical additives including anticoagulant, thrombin, or calcium chloride before applying to the surgical site [9]. PRF is the second generation of APCs originally proposed by Choukroun et al. [10] based on the PRP. It is platelet-rich fibrin with a simple preparation process without adding any chemical additives. CGF is the latest generation of APCs introduced by Sacco in 2006 [11]; it is concentrated by varying the centrifuge speed [12]. In addition, CGF is produced without the addition of any exogenous products and is therefore free from cross-contamination [13].

Recently, APC has gradually attracted the attention of scholars in the treatment of gingival recessions. However, the additional effect of APCs remains controversial. Luo et al. [14] in 2015 assessed the supplementary role of APCs with CAF in the treatment of gingival recessions. Their meta-analysis results showed significant improvement in RD and KTW. The combined application of APCs also had a positive effect on soft tissue healing postoperative. They deemed that APCs could exert a positive impact on CAF. On the contrary, a review by Del Fabbro et al. [15] in 2011 evaluated the adjuvant role of APCs in the prevention of gingival recessions. The outcomes showed no significant improvement in RC and KTW with APCs. Another meta-analysis by Vittorio Moraschini et al. [16] in 2016 evaluated the effects of PRF membranes on the outcomes of clinical treatments in patients with Classes I and II gingival recessions. Their results indicated there was no difference in improving RC, KTW, and CAL with or without APCs.

In summary, the current clinical evidence is still unclear for practitioners. Considering these controversial options, it is our aim to supplement and update the understanding of the role of APCs. The present meta-analysis aims to systematically evaluate whether the three generations of APCs could provide additional effect to CAF for gingival recessions, thus to provide guidance to practitioners in their clinical work.

## 2. Materials and Methods

### 2.1. Focused PICOS Question

This meta-analysis was conducted and reported according to the PRISMA (Preferred Reporting Project Guidelines for Systematic Review and Meta-analysis) protocols [17]. The following statements were used to conduct a systematic search.

The participants (P) included system healthy adults with Class I or II gingival recessions; the intervention (I) was the APCs combined with CAF for gingival recessions; the comparison (C) was conducted with CAF alone; the outcomes (O) comprised clinical parameters including RC, RD, CAL, KTW, and GT; the study (S) was designed for humans and only randomized control trials (RCTs) were enrolled.

### 2.2. Search Strategies

Three electronic databases including Medline, EMBASE, and Cochrane Central Register of Controlled Trials were searched. The search strategy was performed by using the following terms: (“platelet concentrates” OR “platelet-rich plasma” OR “platelet-rich fibrin” OR “concentrated growth factors”) AND (“coronally advanced flap”) AND (“Class I” OR “Class II”) AND (“gingival recessions” OR “root coverage”) AND (“randomized controlled trial”). Only English articles were included. An additional hand search of the following Periodontology journals was performed on the official websites:* Journal of Periodontology*,* Journal of Clinical Periodontology*,* Journal of Dental Research*,* Journal of Dentistry* and* Journal of Periodontal Research*. In addition, the bibliographies of all selected articles and relevant reviews were also searched for missing articles. In addition, grey literature was obtained from Google Scholars (https://xue.glgoo.org/). Unpublished and ongoing trials were obtained from the trial registries (EU Clinical Trials Register: https://www.clinicaltrialsregister.eu). The final search was conducted on May 15, 2019.

### 2.3. Inclusion and Exclusion Criteria

*Inclusion Criteria.* (1) Randomized clinical trials on healthy patients aged 18~60 years old.

(2) Maxillary or mandibular anterior and premolar teeth with Miller's Class I or II gingival recessions confirmed by radiographic and clinical evidence.

(3) Ability to maintain good oral hygiene (O'Leary plaque score [18] ≤20%).

(4) Recession depth ≥2.0 mm, gingival thickness ≥0.5 mm, the width of keratinized gingival ≥2.0 mm after scaling, and root planning.

(5) The only difference between the control and experimental group being that the latter was supplemented with APC.

(6) The language of publication being English.

*Exclusion Criteria*. (1) Pregnancy or lactation for women.

(2) Individuals allergic to medications.

(3) Smoking or use of alcohol or narcotic drugs.

(4) Using drugs that effect periodontal healing such as corticosteroids or calcium channel blockers.

### 2.4. Study Selection and Data Extraction Process

Two reviewers (Rong Li, Yanqing Liu) independently screened the titles and abstracts of the articles retrieved. The same authors also performed the full-text reading of possible relevant articles. If there was any objection, the senior reviewer (Dongmei Zhang) was consulted. Publications that did not meet the inclusion criteria were excluded, and the reasons for exclusion were recorded.

The mean values and standard deviation were collected in an excel sheet by two independent reviewers (Jingya Hou, Yun Wu). The following characteristics of the studies were also extracted, including author, publication year, study design, duration, number of patients and sites, sex, mean age of the patients, smoking, tooth type, site of recessions, and intervention.

### 2.5. Data Items

The primary outcome measures were as follows.

(1) Gingival recessions that attained RC (the percentage of RC was calculated by the following formula [19]:

Percentage of root coverage = ([Preoperative RD - Postoperative RD]/ Preoperative RD)×100%).

(2) Change in RD was expressed as a reduction in recession at the final evaluation (RD was measured at the mid-buccal from CEJ to the gingival margin).

The secondary outcome measures were as follows.

(1) Change in CAL expressed as CAL gain at the final evaluation (CAL referred to the distance from the CEJ to the most apical part of the sulcus).

(2) Change in KTW expressed as KTW gain at the final evaluation (KTW referred to the distance from the mucogingival junction to the free gingival margin).

(3) Change in GT expressed as GT gain at the final evaluation (GT was measured 3 mm below the gingival margin at the attached gingival).

### 2.6. Methodological Quality Assessment

Two individuals (Tong Xu, Haijiao Zhao) independently assessed the methodological quality of each selected study according to the standard for evaluating the risk of bias in the Cochrane Handbook for Systematic Reviews of Interventions (Version 5.1.0) [20]. Seven main quality criteria were examined: (1) random sequence generation method; (2) allocation concealment; (3) blinding of participants and personnel; (4) blinding of outcome assessors; (5) incomplete outcome data; (6) selective outcome reporting; and (7) other bias. All the parameters were assessed as adequate (yes), unclear, or inadequate (no) [21]. When a discrepancy occurred, the discussion was made to reach an agreement. After the quality assessment, the studies were classified into the following categories: (1) low risk: all criteria were met or one criterion was unclear/not met; (2) moderate risk: two criteria were unclear/not met; (3) high risk: more than two criteria were not met. Quality assessment across studies was presented in the form of a graph. According to the Cochrane handbook, Chi-square and Higgins index (*I*^*2*^) were used to judge whether there was heterogeneity.

### 2.7. Data Analysis

The software Revman5.3 (Review Manager version 5.3; The Cochrane Collaboration, Copenhagen, Denmark) was used for meta-analysis. The continuous data (including RC, RD, CAL, KTW, and GT) were expressed as mean difference (MD) and 95% confidence interval (CI), with* P*<0.05 being statistically significant. When the homogeneity between the studies was good (*P*≥0.10,* I*^*2*^≤ 50%), the fixed-effect model was used for meta-analysis. When significantly heterogeneity existed between the studies (*P*<0.10,* I*^*2*^> 50%), the random-effects models were used. The heterogeneity across studies in RC, RD, CAL, KTW, and GT was compared through subgroup analysis. The results of our meta-analysis and responding publication bias were summarized in the forest and funnel plots, respectively.

## 3. Results

### 3.1. Description of Enrolled Studies

**Figure 1:**
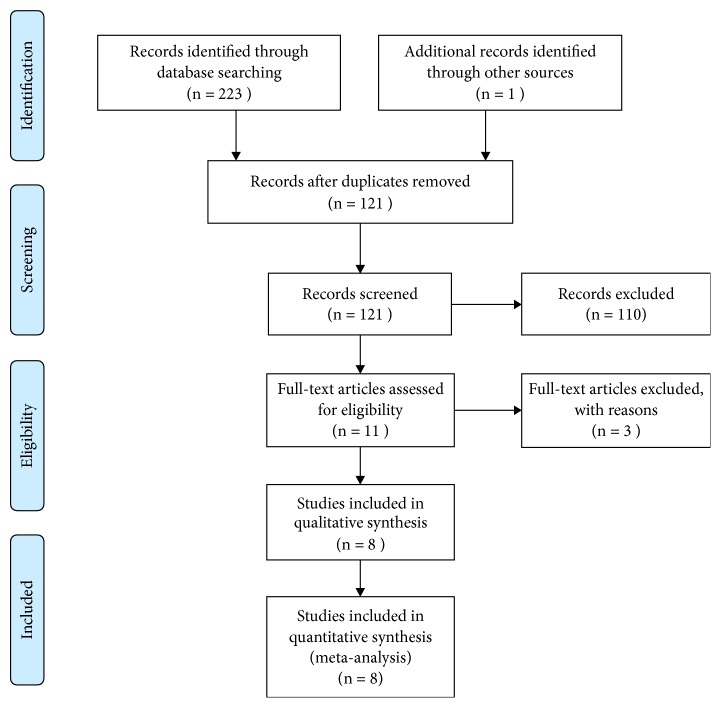
PRISMA flow diagram illustrating the selection process.

The initial electronic search provided 224 papers. Only one study was identified by the hand searching. After duplicates removal, 121 records were screened. After reviewing the titles and abstracts, 14 articles were about APCs for Classes I and II gingival recessions, and the rest 110 papers were excluded. After reviewing the full-text, 3 papers were excluded from the full-text evaluation. The reasons for exclusion were as follows: case series [22, 23] and no control group [24]. The selection process was summarized in Figure 1.

Finally, 8 RCTs [25–32] were selected in our meta-analysis. A total of 170 patients with 328 gingival recessions sites (166 test and 162 control sites) under treatment were enrolled. Three articles adopted a parallel design and five articles adopted a split-mouth design. The characteristics of the included papers were summarized in Table 1. The data of the included studies were extracted in Table 2.

### 3.2. Quality of Studies

**Figure 2:**
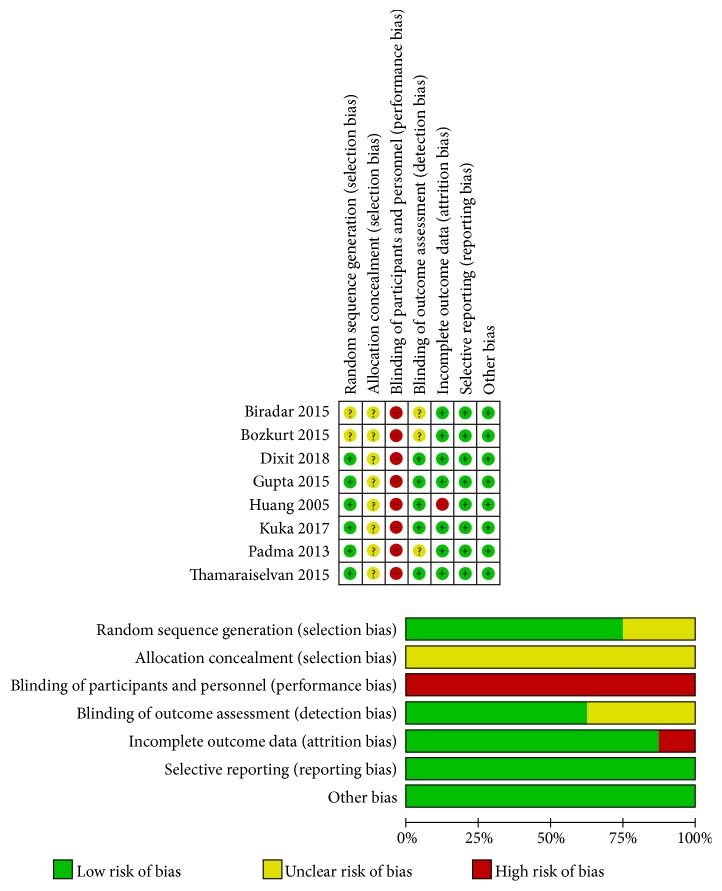
Risk of bias summary of the included studies.

The quality assessment of the selected studies was presented in Figure 2. Sequence generation was reported by six articles: four articles used a coin tossing [26, 29, 31, 32] and two [25, 27] used envelopes; the remaining two articles [28, 30] did not explain the methods of random generation. All enrolled articles did not report allocation concealment which was considered an uncertain risk of bias. Concerning the surgery process, it was impossible to be blind to the personnel. Four articles [25, 27, 31, 32] were blind to the assessors: one article [29] blind both to the patients and the assessor while the remaining three articles were unclear. Follow-up reports were completed for all papers, except one article [25] reporting that one subject in the experiment group dropped out of the study after a 1-month follow-up. No selective reporting and other biases were found. After the evaluation, four articles [27, 29, 31, 32] were classified as moderate risk (two criteria were not met or unclear) and four [25, 26, 28, 30] as high risk (three or four criteria were not met or unclear).

### 3.3. Additional Effect of APCs

**Figure 3:**
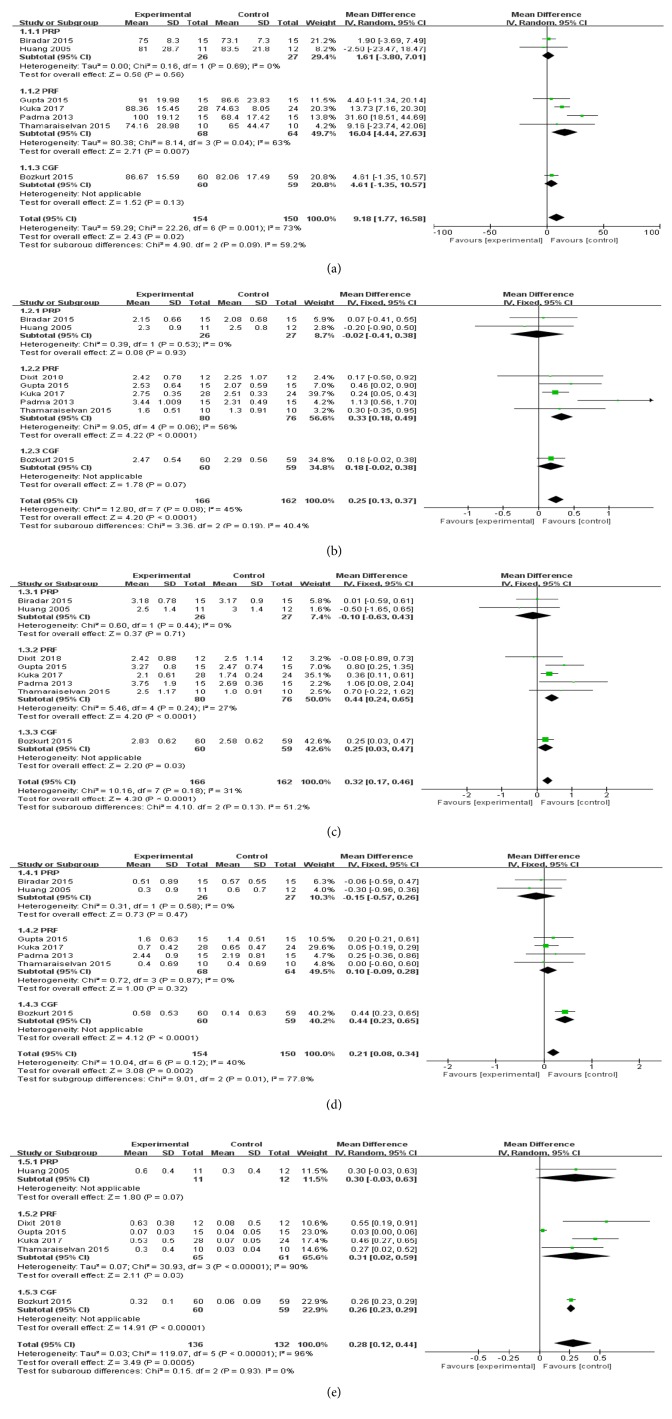
Forest plots for RC change (a), RD reduction (b), CAL gain (c), KTW gain (d), and GT gain (e).

*RC gain*: altogether, seven articles [25–31] were analyzed. Dixit et al.'s article was excluded from the analysis because it did not report specific data. The random-effects model was conducted due to its high heterogeneity (*I*^2^=73%). The results of the analysis showed that APCs exerted a greater RC gain when added to CAF compared with CAF alone (Figure 3(a)). In the subgroup analysis of PRF, there was significant difference between the test and the control groups in RC, with an MD of 16.04 mm (95% CI: 4.44~27.63 mm;* P*=0.007), while PRP subgroup and CGF subgroup showed no significant differences between the two groups.

*RD reduction*: meta-analysis was conducted among all the eight records. A fixed-effects model was applied (*I*^2^=45%). In terms of the results, the APCs group showed more RD reduction compared with CAF alone (Figure 3(b)). A beneficial effect with an MD of 0.33 mm (95%CI: 0.18~0.49 mm;* P*<0.001) was found in the subgroups of PRF. In contrast, no significant RD reduction was shown in subgroups of PRP and CGF.

*CAL reduction*: meta-analysis was performed in 8 studies. A fixed-effects model was used (*I*^2^=31%). The results showed that the use of APCs determined a significant gain of CAL when added to CAF in the treatment of gingival recessions (Figure 3(c)). Statistical differences between the test and the control in the subgroup of PRF and CGF were found, with an MD of 0.44 mm (95%CI: 0.24~0.65 mm;* P*<0.001) and an MD of 0.25 mm (95%CI: 0.03~0.47 mm;* P*=0.03), respectively. No difference was found in the subgroup of PRP.

*KTW gain*: The meta-analysis of keratinized tissue increasing was performed on seven studies. Dixit et al.'s article was excluded from the analysis because it did not provide specific data. A fixed-effects model was used (*I*^2^=40%). The results of the included studies showed that the use of APCs determines a greater KTW gain than CAF alone (Figure 3(d)). From the results of the subgroup analysis, only CGF membrane could significantly improve the KTW, with an MD of 0.21 mm (95%CI: 0.08~0.34 mm;* P*<0.001). The PRP and PRF failed to show any improvement.

*GT gain*: two records [26, 30] were excluded from our meta-analysis for its incomplete data. The heterogeneity was high (*I*^2^=96%), so a random-effects model was used. The results of our meta-analysis showed that APCs groups obtained a greater GT gain than CAF alone groups (Figure 3(e)). Subgroup analysis revealed significant differences for PRF and CGF, with an MD of 0.31 mm (95%CI: 0.02~0.59 mm;* P*=0.03) and an MD of 0.26 mm (95%CI: 0.23~0.29 mm;* P*<0.001), respectively. Nevertheless, no significant difference was found in the subgroup of PRP.

### 3.4. Sensitivity Analyses

**Figure 4:**
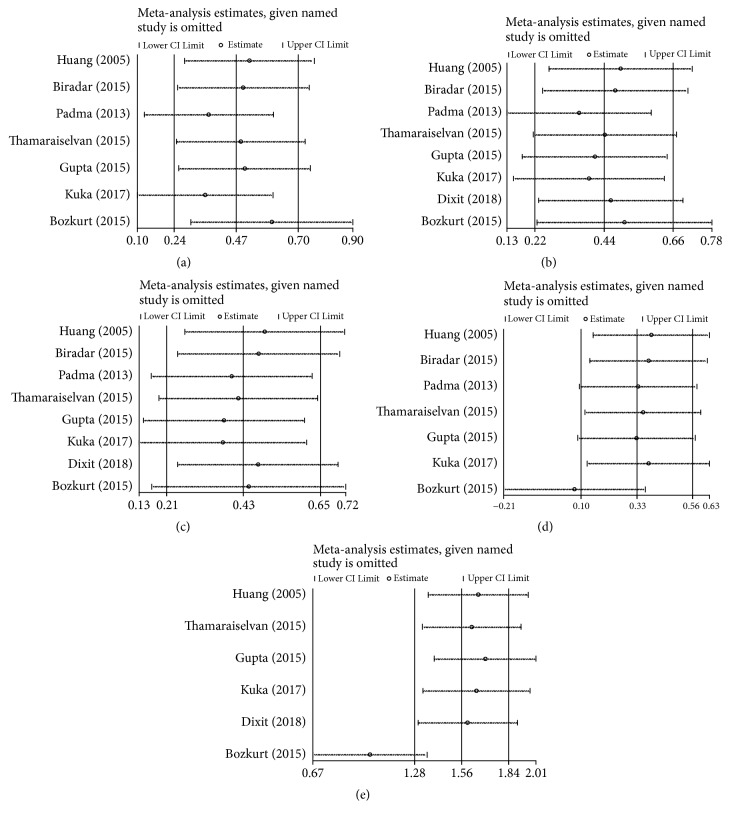
Sensitivity analysis was applied by comparing CAF+PRP/PRF/CGF with CAF alone. (a) RC change; (b) RD reduction; (c) CAL gain; (d) KTW gain; (e) GT gain.

Sensitivity analyses were investigated by discarding one research every time to assess the impact of single research on the general outcomes. Except excluding the research of Bozkurt et al. [28], the overall stability of our results was shown in Figures 4(d) and 4(e).

### 3.5. Publication Bias

**Figure 5:**
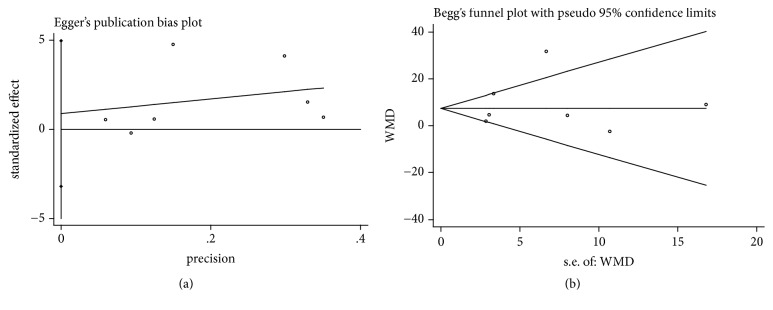
Publication bias of RC change was applied by comparing CAF+PRP/PRF/CGF with CAF alone. (a) Egger's linear regression; (b) Begg's funnel plot.

**Figure 6:**
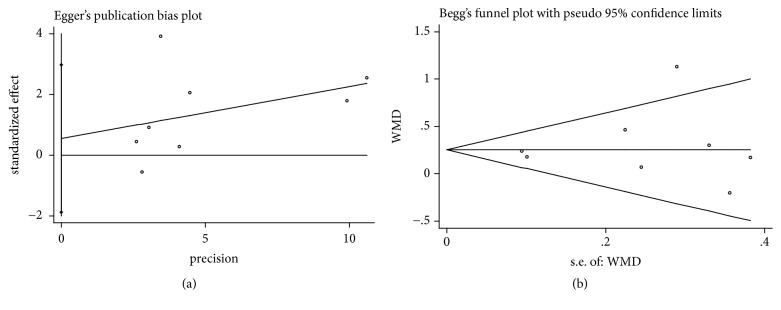
Publication bias of RD reduction was applied by comparing CAF+PRP/PRF/CGF with CAF alone. (a) Egger's linear regression; (b) Begg's funnel plot.

**Figure 7:**
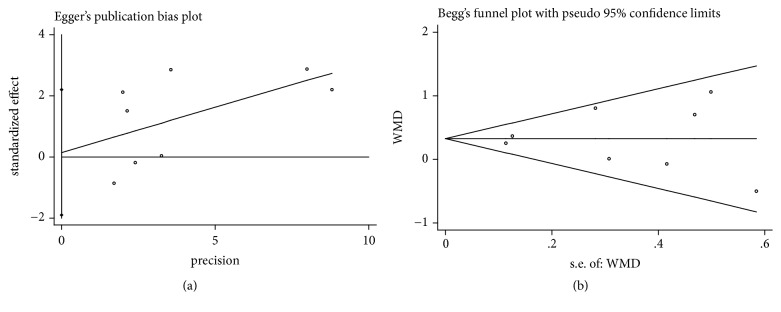
Publication bias of CAL gain was applied by comparing CAF+PRP/PRF/CGF with CAF alone. (a) Egger's linear regression; (b) Begg's funnel plot.

**Figure 8:**
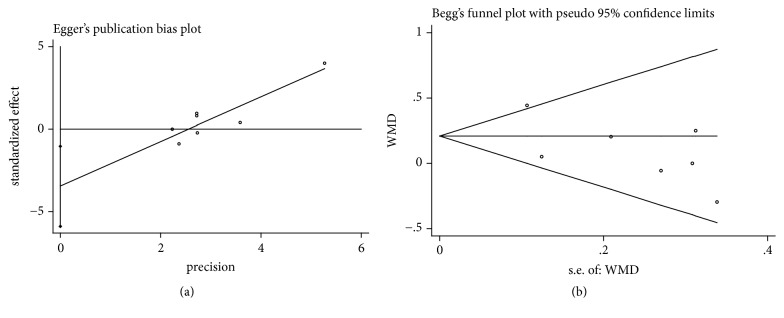
Publication bias of KTW gain was applied by comparing CAF+PRP/PRF/CGF with CAF alone. (a) Egger's linear regression; (b) Begg's funnel plot.

**Figure 9:**
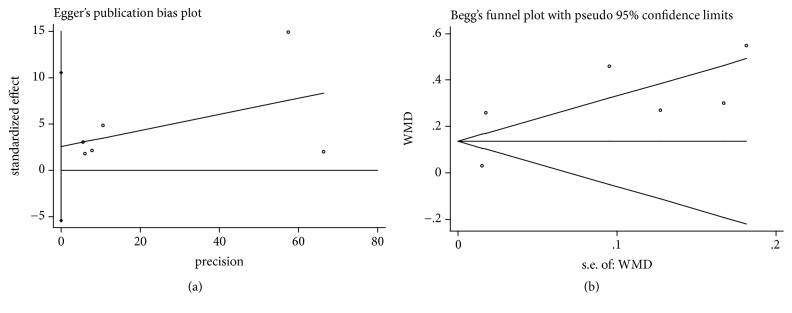
Publication bias of GT gain was applied by comparing CAF+PRP/PRF/CGF with CAF alone. (a) Egger's linear regression; (b) Begg's funnel plot.

It was considered that there was no publication bias by the Begg's and Egger's test (*P*>0.05), which further supported the reliability of the enrolled studies (Figures 5, 6, 7, 8, and 9).

## 4. Discussion

To achieve safe and effective outcomes for gingival recessions, researchers have done numerous works to improve CAF surgical procedures, including the usage of regeneration materials. As APCs can be easily obtained and it can promote wound healing and minimize the occurrence of infection, the additional effect of APCs to CAF for gingival recessions has been investigated. However, there was a lack of clinical evidence to confirm this effect. The present meta-analysis was intended to assess the adjunctive efficacy of three types of APCs when combined with CAF for the treatment of Classes I and II gingival recessions.

On the whole, the results of the present meta-analysis showed the three types of APCs had beneficial effects in all effect sizes compared with CAF alone. Regarding the primary outcomes RC and RD, only PRF and CGF showed significant differences compared with the CAF alone. It seemed that PRP would not bring a significant difference in primary outcomes. The heterogeneity of RC seems to be related to the studies of Padma and Kuka. A modified CAF (full and split flap design were combined) was used in Padma's study other than the traditional CAF. And the duration of Kuka's study was 12 months, while the duration was 6 months in other studies. We believed that the different surgical method and duration might lead to the heterogeneity. Actually, after excluding these two studies, our subgroup analysis found that the heterogeneity was low (*I*^*2*^ = 0%). As to the secondary outcomes of CAL, KTW, and GT, PRF and CGF showed significant results in CAL and GT. Only CGF showed a significant result in KTW. PRP showed no significant results in any secondary outcomes. The heterogeneity of GT seems to be related to the study of Bozkurt because of the different prepare process of CGF. In fact, the results of our subgroup analysis found that* I*^2^ was 0% after excluding this one.

According to the subgroup analysis results, the three types of APCs did not have the same therapeutic effects on gingival recessions in the CAF procedure. The results of PRP subgroup analysis, especially, showed no significant difference in all the outcome variables. This result was in accordance with Keceli et al. [19]. They compared the CTG+PRP and the CTG alone in the treatment of Miller's I/ II recessions. Their results showed PRP provides no additional benefits to CTG in terms of RC, RD, CAL, and KTW at the 6- and 12-month follow-up. The reasons could be ascribed as follows: First, as the first generation of platelet products, there were certain limitations and deficiencies in the production process of PRP. The preparation of PRP required the artificial addition of thrombin and other preparations. The resulting fibrin had a dense four-molecule structure with small interfibrous pores, which was not conducive to the attachment of cytokines and proliferation of cells [33]. Secondly, PRP released a series of growth factors for 7 days, which peaked on the first day of application [34]. The growth factors in PRP cannot be supplied stably and continuously. But controversially, many previous studies reported that PRP had the ability to promote bone regeneration and soft tissue repair [35–37]. As we know, CAF is one of the safe and effective surgical methods to treat gingival recessions, the clinical effects of it alone in the gingival recessions have been reported in a previous review [38]. We considered the function of PRP might be counteracted by the CAF. On the other hand, based on the limited two studies, our results of PRP need to be further confirmed by more researches.

The results of PRF showed that it had a wide range of effects, including significant improvement on RC, RD, CAL, and GT. The concentration of growth factors and matrix proteins in PRF is higher than PRP [33]. In addition, the fibrin of PRF has a three-dimensional structure. Such a fibrin network will lead to more efficient cell migration and proliferation and can protect the growth factors from proteolysis, thus lengthening the release of growth factors, prolonging the duration of action and promoting tissue healing [7, 39]. Scholar's molecular research has shown PRF can continuously release growth factors within 21 days and peak in 7 days [40]. Dohan Ehrenfest [41] and Zumstein [42] compared the PRP and PRF membranes in vitro. They found the two gels presented 2 very different profiles: the PRP released its most growth factors in the first hours and completely dissolved after 3 days. In contrast, the PRF membrane remained solid and intact after 7 days and continuously released numerous growth factors and matrix molecules. Another superiority of PRF over PRP lies on that PRF fibers aggregate a large number of white blood cells, which have anti-inflammatory and antibacterial effects [43, 44]. Among the four included PRF records, both the test and the control groups had a mild gain of KTW between baseline and 6 months. Nevertheless, the difference of intergroup was statistically nonsignificant (MD: 0.10 mm; 95%CI: -0.09~0.28 mm;* P*=0.32), which is in line with the results of Del Fabbro et al. [15]. Their meta-analysis of platelet concentrates for keratinized tissue enhancement showed no significant effect (MD: 0.18 mm; 95% CI: -0.19 ~ 0.54 mm;* P* = 0.34).

Only one record [28] on CGF was selected in this analysis. The subgroup analysis of CGF showed no significant difference in RC and RD, while there were some positive effects in CAL, KTW, and GT. Keratinized gingival is attached to the root surface or underlying bone; the presence of keratinized gingival is an important aspect for the maintenance of gingival health and the prevention of periodontal disease progression [45]. The reduction of gingival thickness can lead to periodontal attachment loss and marginal tissue recession, which is a major concern for periodontal disease progression [46]. Teeth with sufficient KTW and GT are more resistant to inflammation or trauma [47]. In other words, the augmentation of KTW and GT may enhance the long-term predictability of CAF surgery through reducing postoperative recurrence and provide long-term stability [48]. On the other hand, since only one study was qualified to be enrolled, the results of CGF analysis were limited and could not be used universally. The benefits of CGF as an adjunction to gingival recessions remain questionable. We expect more researches to be conducted in this area.

At present, several meta-analyses on APCs for intrabony defects, maxillary sinus elevation, and furcation defects had been published. However, unlike intrabony defects or furcation defects, there is a significant soft tissue defect when the gingival recession occurs, and the available tissue engineering treatment is limited. Different from the existing articles on the application of APCs for gingival recessions, we choose the simplest surgical method (CAF) without other confounding factors like reconstructive techniques, to minimize the clinical heterogeneity caused by the surgical method. Furthermore, unlike other articles, we set the RC as the primary outcome. This outcome variable is more suitable for evaluating gingival recessions because the ultimate goal of gingival recessions treatment is to gain the coverage of root surface and to obtain an optimal aesthetic outcome. To the best of our knowledge, this is the first meta-analysis that included the third generation of APCs (named CGF), which helps us to have a more comprehensive understanding of APCs in the treatment of gingival recessions.

However, there were also some limitations to this meta-analysis. Firstly, there was an inherent heterogeneity between the included articles. Two articles [25, 31] were Class I gingival recessions and the remaining six were Classes I and II recessions. One article [30] included molar teeth and four articles [25, 27, 29, 31] included anterior and premolar teeth. Due to the limited studies that could be searched, the entire Class I and Class II gingival recessions, anterior teeth, premolar teeth, and molar teeth were considered together, whereas the depth of the recession defects and the types of teeth can also affect the prognosis of regenerative surgeries. All these taken together may be drawbacks in this analysis. Other heterogeneities should also be considered in future clinical studies, including patient populations, methods of preparation of APCs, and duration of follow-up. Another limitation of this paper was the high risk of bias in the selected studies. Because of the different production processes of APCs, it was impossible to conduct the allocation concealment strictly or to be blind to the personnel. Our risk of bias assessment results showed that among 8 included studies, 4 studies were classified as moderate risk, and 4 as high risk. Taken together, the conclusions of our analysis are limited, and more studies with low risk of bias in this field are needed in the future to provide definitive clinical guidance for periodontal treatment.

## 5. Conclusions

After the system review and meta-analysis, we can initially get the following conclusions:.

(1) According to the results of our meta-analysis which enrolled five studies, PRF could exert additional effect to CAF. We considered that PRF should be preferred for the treatment of Classes I and II gingival recessions.

(2) Based on the limited studies, it seemed that PRP failed to show any additional effect when combined with CAF. It was not suggested for the therapy of Classes I and II gingival recessions, so as to alleviate the preoperative pain of patients.

(3) Given the limited research and high risk of bias, it is still needed to confirm the additional effect of CGF by more high-quality studies.

(4) Overall, the risk of bias of the articles included in APCs was high, and more low-risk and high-quality researches were needed.

## Figures and Tables

**Table 1:** General information of enrolled articles.

References (year)	Study Design (Duration)		Population			Miller Class	Site of Recessions	Intervention (No. of sites)	
		Sample size	Sex	Age	Smoking			Test	Control
		(No. of patients)		(Range)	(Yes, No?)				
Huang et al. (2005)	RCT/parallel	23 sites (23)	17F/6M	43.8±11.9	No	I	Maxillary or mandibular	CAF + PRP (11)	CAF (12)
	(6 months)						anterior and premolar teeth,		
Padma et al. (2013)	RCT/split-mouth	30 sites (15)	NR	18~35	No	I and II	NR	CAF+PRF (15)	CAF (15)
	(6 months)								
Thamaraiselvan et al.	RCT//parallel	20 sites (20)	2F/18M	21~47	No	I and II	Maxillary or mandibular	CAF+PRF (10)	CAF (10)
(2015)	(6 months)						anterior and premolar teeth,		
Bozkurt et al. (2015)	RCT/split-mouth	119 sites (20)	13F/7M	37.10±1.03	No	I and II	NR	CAF+CGF (60)	CAF (59)
	(6 months)								
Gupta et al. (2015)	RCT/split-mouth	30 sites (26)	NR	20~50	No	I and II	Maxillary anterior and	CAF+PRF (15)	CAF (15)
	(6 months)						premolar teeth,		
Biradar et al. (2015)	RCT/ parallel	30 sites (30)	NR	18~45	No	I and II	Maxillary or mandibular anterior,	CAF+PRP (15)	CAF (15)
	(4 months)						premolar and molar teeth,		
Kuka et al. (2017)	RCT/split-mouth	52 sites (24)	13F/11M	32.35±6.41	No	I	Maxillary or mandibular	CAF+PRF (28)	CAF (24)
	(12 months)						anterior and premolar teeth,		
Dixit et al. (2018)	RCT/split-mouth	24 sites (12)	5F/7M	18~50	No	I and II	NR	CAF+PRF (12)	CAF (12)
	(6 months)								

NR: Not Reported CAF: Coronally Advanced Flap PRP: Platelet-Rich Plasma PRF: Platelet-Rich Fibrin CGF: Concentrated Growth Factor.

**Table 2:** The data of the included articles.

References(Year)	*MD *in *PD *Between Baseline and Final Follow-Up (mm)	*MD *in *CAL *Between Baseline and Final Follow-Up (mm)	*MD *in *RC *Between Baseline and Final Follow-Up (%)	*MD *in *KMW *Between Baseline and Final Follow-Up (mm)	*MD *in *GT *Between Baseline and Final Follow-Up (mm)	*MD *in *RD *Between Baseline and Final Follow-Up (mm)	Other Outcomes
Huang et al. (2005)	NR	2.5 ± 1.4(T)	81.0 ± 28.7 (T)	0.3 ± 0.9 (T)	0.6± 0.4 (T)	2.3 ± 0.9 (T)	RW PI GI
	NR	3.0 ± 1.4 (C)	83.5 ± 21.8 (C)	0.6 ± 0.7 (C)	0.3± 0.4 (C)	2.5± 0.8 (C)	
Padma et al. (2013)	NR	3.75 ± 1.90 (T)	100 ± 19.12 (T)	2.44 ± 0.90 (T)	NR	3.44 ± 1.009 (T)	RD
	NR	2.69 ± 0.36 (C)	68.40 ± 17.42 (C)	2.19 ± 0.81 (C)	NR	2.31 ± 0.49 (C)	
Thamaraiselvan et al. (2015)	0.40 ± 0.51 (T)	2.50 ± 1.17 (T)	74.16 ± 28.98 (T)	0.40 ± 0.69 (T)	0.30 ± 0.10 (T)	1.60 ± 0.51 (T)	RW CRC PI GI
	0.30± 0.48 (C)	1.80 ± 0.91 (C)	65.00 ± 44.47 (C)	0.40 ± 0.69 (C)	0.03 ± 0.04 (C)	1.30 ± 0.91 (C)	
Bozkurt et al. (2015)	0.37 ± 0.49 (T)	2.83 ± 0.62 (T)	86.67 ±1 5.59 (T)	0.58 ± 0.53 (T)	0.32 ± 0.10 (T)	2.47 ± 0.54 (T)	RW CRC
	0.29 ± 0.46 (C)	2.58 ± 0.62 (C)	82.06 ± 17.49 (C)	0.14 ± 0.63 (C)	0.06 ± 0.09 (C)	2.29 ± 0.56 (C)	
Gupta et al. (2015)	0.73 ± 0.46 (T)	3.27 ± 0.80 (T)	91.00 ± 19.98 (T)	1.60 ± 0.63 (T)	0.07 ± 0.03 (T)	2.53 ± 0.64 (T)	
	0.41 ± 0.51 (C)	2.47 ± 0.74 (C)	86.60 ± 23.83 (C)	1.40 ± 0.51 (C)	0.04 ± 0.05 (C)	2.07 ± 0.59 (C)	
Biradar et al. (2015)	0.90 ± 1.68 (T)	3.18 ± 0.78 (T)	75.00 ± 8.30 (T)	0.51 ± 0.89 (T)	NR	2.15 ± 0.66 (T)	RW
	0.87 ± 0.32 (C)	3.17 ± 0.90 (C)	73.10 ± 7.30 (C)	0.57 ± 0.55 (C)	NR	2.08 ± 0.68 (C)	
Kuka et al. (2017)	0.65 ± 0.24 (T)	2.10 ± 0.61 (T)	88.36 ± 15.45 (T)	0.70 ± 0.42 (T)	0.53 ± 0.05 (T)	2.75 ± 0.35 (T)	RW PI GI BOP CRC
	0.78 ± 0.34 (C)	1.74 ± 0.24 (C)	74.63 ± 8.05 (C)	0.65 ± 0.47 (C)	0.07 ± 0.05 (C)	2.51 ± 0.33 (C)	
Dixit et al. (2018)	NR	2.42 ± 0.88 (T)	NR	NR	0.63 ± 0.38 (T)	2.42 ± 0.78 (T)	SBI RW
	NR	2.50 ± 1.14 (C)	NR	NR	0.08 ± 0.50 (C)	2.25 ± 1.07 (C)	

T: Test Group C: Control Group *MD: *Medium Difference PD: Probe Depth CAL: Clinical Attachment Level RC: Root Coverage KTW: Keratinized Tissue Width GT: Gingival Thickness.

RD: Recession Depth NR: Not Reported RW: Recession Width PI: Periodontal Index GI: Gingival Index CRC: Completely Root Coverage BOP: Bleeding on Probing SBI: Sulcus Bleeding Index.
